# Comparative evaluation of apical root resorption in adult patients treated with clear aligners and fixed orthodontic appliances using CBCT

**DOI:** 10.2340/aos.v85.46274

**Published:** 2026-06-11

**Authors:** Rakhi Issrani, Abdullah Hammad Alshammari, Mohammad Raji Alrwuili, Mohammed Ahmed Aljabab, Nasser Attallah Alenzi, Poonam Joshi, Yousef T. Aldoseri

**Affiliations:** a Department of Preventive Dentistry, College of Dentistry, Jouf University, Sakaka, Kingdom of Saudi Arabia; b Qurayyat Specialized Dental Center, Al-Qurayyat, Kingdom of Saudi Arabia; c Western Riyadh Dental Complex, Riyadh, Kingdom of Saudi Arabia; d Department of Conservative Dentistry and Endodontics, Dr. D.Y Patil Dental College and Hospital, Dr. D.Y Patil Vidyapeeth, Pimpri, Pune, India; e Department of Dentistry, Riyadh Elm University, Riyadh, Kingdom of Saudi Arabia

**Keywords:** Root resorption, orthodontic appliances, cone-beam computed tomography, orthodontics

## Abstract

**Background:**

Orthodontically induced external apical root resorption is a common iatrogenic effect of tooth movement. With the increasing use of clear aligner therapy, it is important to evaluate whether its biomechanics reduce root resorption compared with fixed orthodontic appliances.

**Aim:**

To compare apical root resorption in patients treated with clear aligners and fixed appliances using cone-beam computed tomography (CBCT).

**Methodology:**

A retrospective comparative CBCT-based analysis was conducted on 120 orthodontic patients. Root volumes and lengths were measured from the cementoenamel junction to the apex using pre- and post-treatment CBCT scans. Apical root resorption was quantified as percentage volume loss and absolute root length reduction. Primary analyses employed linear mixed-effects models adjusted for clustering of teeth within patients and for tooth type, arch, extraction status, and treatment duration.

**Results:**

Clear aligner therapy was associated with significantly lower apical root resorption than fixed appliances. Mean percentage root volume loss was lower in the aligner group (6.1 ± 4.8%) than in the fixed appliance group (8.4 ± 6.1%) (*p* = 0.004). Mean root length reduction was also smaller with aligners (*p* = 0.001). Clinically significant root resorption occurred less frequently in the aligner group (*p* = 0.048). Longer treatment duration increased resorption risk.

**Conclusion:**

Clear aligner therapy is associated with less apical root resorption than fixed orthodontic treatment, while treatment duration remains an independent risk factor.

## Introduction

Orthodontically induced external apical root resorption (OIEARR) is a well-recognized iatrogenic consequence of orthodontic tooth movement and remains a major biological concern during treatment planning and risk assessment. Although most cases are mild and clinically insignificant, moderate-to-severe resorption can compromise root integrity, affect long-term tooth prognosis, and influence treatment outcomes. The severity of OIEARR is known to vary considerably among individuals and is influenced by multiple factors, including force magnitude and duration, tooth type, treatment mechanics, and individual biological susceptibility [1, 2].

Accurate assessment of apical root resorption is essential for meaningful comparison between orthodontic treatment modalities. Conventional two-dimensional radiographic techniques, such as panoramic and periapical radiographs, have inherent limitations related to distortion, magnification, and superimposition, which may underestimate the extent of root resorption [3]. Cone-beam computed tomography (CBCT) enables three-dimensional evaluation of root morphology and allows both linear and volumetric quantification of resorptive changes, making it a valuable tool for detailed assessment in clinical research [4, 5].

The increasing use of clear aligner therapy as an alternative to conventional fixed orthodontic appliances has prompted interest in its potential biological effects on dental and periodontal tissues. Clear aligners are generally designed to deliver lighter and more intermittent forces compared with the relatively continuous forces applied by fixed appliances. It has therefore been hypothesized that aligner-based mechanics may reduce the risk or severity of apical root resorption, particularly in teeth subjected to greater orthodontic loading, such as incisors [6]. Several clinical and CBCT-based studies have reported lower levels of root resorption with aligner therapy [7–9]; however, findings remain inconsistent due to variability in study design, sample size, outcome measures, and statistical handling of clustered tooth-level data [10].

Despite growing interest in this topic, high-quality CBCT-based comparative studies with adequate sample sizes and appropriate multilevel statistical modeling remain limited. Many previous investigations have not simultaneously assessed volumetric and linear root changes or adequately adjusted for confounding factors such as tooth type, treatment duration, and extraction status. Therefore, the present study aimed to perform a CBCT-based comparative evaluation of apical root resorption in adult patients treated with clear aligner therapy and fixed orthodontic appliances, using mixed-effects models to account for clustering and clinically relevant covariates. The null hypothesis was that there would be no difference in the extent or prevalence of apical root resorption between the two treatment modalities.

## Materials and methods

### Study design

This hospital-based retrospective comparative observational study evaluated pre- and post-treatment CBCT records of orthodontic patients treated with clear aligners or fixed appliances. This study was conducted in accordance with the STROBE guidelines for observational studies. Ethical approval no. HAPO-13-S-001-11454 was received from the Local Committee of Bioethics, Jouf University. Written informed consent for use of radiographic data was obtained from all participants.

### Study population and sample selection

From January 2023 to January 2025, data of patients who underwent orthodontic treatment were collected. The database yielded 312 patient profiles. A total of 120 patients fulfilling the eligibility criteria were included in the final analysis. The inclusion criteria were the following: aged between 18 and 30 years, full permanent dentition, good quality pre- and post-treatment CBCT scans, no pre-existing resorption (pre-existing root resorption was ruled out based on baseline CBCT evaluation) or developmental anomalies (like dilacerations, short root anomaly, and taurodontism). Patients who were smokers, pregnant, had a history of dental trauma, bruxism, incomplete medical records, previous orthodontic treatment, or systemic diseases were excluded.

The study comprised two groups: Group I included patients treated with clear aligners (*n* = 60), and Group II included patients treated with fixed orthodontic appliances (*n* = 60).

### Orthodontic treatment protocols

Patients in the aligner group were treated using a commercially available thermoplastic clear aligner system based on digital treatment planning and staged tooth movement. Aligner staging typically involved incremental tooth movements of approximately 0.25 mm per aligner with aligner replacement every 14 days and recommended daily wear of approximately 22 hours, Composite attachments were placed as needed to facilitate specific tooth movements. Meanwhile, the fixed appliance group was treated and tied up to a pre-adjusted McLaughlin–Bennett–Trevisi prescription bracket (0.022-inch slot) that employed a universal wire sequence ranging from 0.014-inch NiTi through to 0.019 × 0.025-inch stainless steel wires. The duration of active orthodontic treatment for both groups was measured in months from appliance placement (or initiation of aligner therapy) to completion of active treatment, excluding the retention phase.

Although force magnitude could not be standardized retrospectively, both treatment modalities followed manufacturer- and clinician-recommended protocols.

All orthodontic treatments were performed by a single experienced orthodontist, which ensured consistency in treatment planning and biomechanical protocols.

### CBCT acquisition protocol and image processing

All CBCT scans were obtained for clinical diagnostic purposes as part of routine orthodontic assessment, and no additional imaging was performed for research. Before and after treatment, CBCT scans (SCANORA 3Dx, Nahkelantie 160, Tuusula, Finland) were performed by the same device (voxel size 0.20 mm; field of view 16×10 cm; 90 kVp, 10 mA) with the Frankfurt horizontal plane parallel to the floor. DICOM images were processed by OnDemand 3D and individual tooth volumes from cementoenamel junction (CEJ) to root apex were segmented. Segmentation was performed using semi-automated thresholding followed by manual refinement. Root volumes were delineated from the CEJ to the apex in all planes. A pilot segmentation of 20 teeth was used to standardize examiner calibration. The voxel-based superimposition was achieved to superimpose pre- and post-treatment images, and the volume (mm³) and length (mm) of resorption root apex were calculated.

### Measurement reliability

Two trained oral and maxillofacial radiologists conducted the measurements and intraclass correlation coefficient (ICC) was calculated for intra- and inter-examiner reliability. CBCT-based root volume and length measurements demonstrated excellent intra- and inter-examiner reliability (ICC > 0.93).

### Statistical analysis

Data were analyzed using SPSS version 22.0 (IBM Corp., Armonk, NY, USA). Descriptive statistics were used to summarize baseline characteristics and outcomes. Initial unadjusted between-group comparisons were performed using independent *t* tests or Mann–Whitney U tests, as appropriate. To account for multiple teeth assessed within the same patient, mixed-effects models were applied. Linear mixed-effects models were used to evaluate percentage root volume loss, while mixed-effects logistic regression was used to assess clinically significant root resorption (≥ 2 mm or ≥ 15% volume loss). Models were adjusted for tooth type, arch, treatment duration, and extraction status. Intra- and inter-examiner reliability was assessed using intra-class correlation coefficients. A post hoc power analysis indicated that the sample size provided > 80% power to detect a between-group difference of 2% in volumetric root loss. A *p*-value < 0.05 was considered statistically significant.

## Results

Baseline characteristics were largely comparable between the two groups, with no statistically significant differences in age, gender distribution, baseline root morphology, or extraction plan. However, treatment duration was significantly longer in the fixed appliance group (*p* = 0.002) (Table 1).

**Table 1:** Baseline characteristics of study participants.

Characteristic	Clear aligner (*n* = 60)	Fixed appliance (*n* = 60)	Standardized mean differences	*P*
Age, years (mean ± SD)	23.6 ± 5.2	23.9 ± 5.0	0.05	0.75
Gender Female, *n* (%) Male, *n* (%)	38 (63.3) 22 (36.7)	36 (60.0) 24 (40.0)	0.07 0.07	0.85
Treatment duration, months (median [IQR])	11.8 [9.5–14.0]	14.7 [12.2–17.1]	0.53	0.002*
Extraction plan, *n* (%)	10 (16.7)	12 (20.0)	0.08	0.81
Teeth per patient (mean ± SD)	12.0 ± 2.1	12.0 ± 2.0	0.00	1.00
Baseline root volume (mm³, mean ± SD)	320 ± 85	323 ± 88	0.04	0.85
Baseline root length (mm, mean ± SD)	22.9 ± 2.2	23.1 ± 2.3	0.09	0.63
Tooth-type distribution (Incisors/Canines/Premolars/Molars)	28/12/40/20	27/13/40/20	0.03	0.98

* Significant.

Clear aligner therapy was associated with significantly lower apical root resorption compared with fixed appliances (Table 2). This difference was evident for both volumetric loss and root length reduction. The greatest between-group differences were observed in incisors, while posterior teeth exhibited smaller differences.

**Table 2:** Comparison of apical root resorption outcomes between treatment groups.

Outcome	Tooth set	Clear aligner (mean ± SD)	Fixed appliance (mean ± SD)	95% CI	*P*
% volume loss	All teeth	6.1 ± 4.8	8.4 ± 6.1	-2.3 [-3.8, -0.8]	0.004
	Incisors	8.9 ± 5.6	12.2 ± 7.3	-3.3 [-5.4, -1.1]	0.003
	Canines	6.5 ± 4.2	8.0 ± 5.0	-1.5 [-3.2, +0.2]	0.08
	Premolars	5.2 ± 3.6	6.7 ± 4.2	-1.5 [-2.8, -0.1]	0.03
	Molars	3.6 ± 2.7	4.3 ± 3.1	-0.7 [-1.6, +0.3]	0.18
Root length change (mm)	All teeth	-0.55 ± 0.46	-0.79 ± 0.62	+0.24 [+0.10, +0.39]	0.001
≥ 2 mm loss (%)	All teeth	7.9%	11.8%	-3.9 [-7.7, -0.1]	0.048

After adjustment for clustering and relevant confounders, treatment modality and treatment duration emerged as significant predictors of apical root resorption (Table 3). Clear aligner therapy was consistently associated with lower volumetric root loss compared with fixed appliances, while longer treatment duration was associated with increased resorption irrespective of appliance type. Incisors demonstrated the greatest susceptibility to resorption, whereas premolars and molars showed significantly lower resorption compared with incisors. For each additional 6 months of active treatment, percentage root volume loss increased by approximately 0.7%. Age, gender, arch type, and extraction plan were not statistically significant predictors, although extraction cases demonstrated a trend toward higher resorption. ICC indicated moderate clustering at the patient and tooth levels, supporting the use of mixed-effects modeling.

**Table 3:** Linear mixed-effects model for percentage root volume loss.

Predictor	β	SE	95% CI	*P*
Treatment (Fixed appliance versus Clear aligner)	-1.9	0.68	-3.2, -0.6	0.005
Age (per 10 years)	+0.2	0.25	-0.3, +0.7	0.42
Gender (ref: female)	+0.1	0.28	-0.4, +0.6	0.68
Tooth type (ref: Incisor) Canine Premolar Molar	-0.8 -1.5 -2.3	0.52 0.49 0.56	-1.8, +0.2 -2.4, -0.6 -3.4, -1.1	0.12 0.001 < 0.001
Arch (ref: maxillary)	-0.5	0.31	-1.1, +0.1	0.10
Duration (per 6 months)	+0.7	0.20	+0.3, +1.1	0.001
Extraction plan (yes)	+0.9	0.57	-0.2, +2.0	0.10
Model fit	ICC_patient = 0.19 ICC_tooth = 0.16			

Multivariable mixed-effects logistic regression analysis demonstrated that treatment modality and treatment duration were significant predictors of clinically significant apical root resorption (≥ 2 mm) (Table 4). Compared with clear aligner therapy, fixed orthodontic appliances were associated with higher odds of clinically significant root resorption (adjusted odds ratios [aOR] = 0.62; 95% confidence interval [CI]: 0.3–0.9; *p* = 0.03). Tooth type significantly influenced resorption risk, with premolars (aOR = 0.59; 95% CI: 0.3–0.9; *p* = 0.03) and molars (aOR = 0.47; 95% CI: 0.2–0.8; *p* = 0.02) demonstrating lower odds of clinically significant resorption compared with incisors, while canines did not differ significantly. Longer treatment duration was associated with increased odds of clinically significant root resorption, with each additional 6 months of active treatment increasing the odds by approximately 25% (aOR = 1.25; 95% CI: 1.0–1.5; *p* = 0.02). Extraction status was not a significant predictor after adjustment.

**Table 4:** Mixed-effects logistic regression model for clinically significant root resorption.

Predictor	Adjusted odds ratios (aORs)	95% CI	*P*
Treatment (Fixed appliance versus Clear aligner)	0.62	0.3–0.9	0.03
Tooth type (ref: Incisor) Canine Premolar Molar	0.91 0.59 0.47	0.5–1.4 0.3–0.9 0.2–0.8	0.69 0.03 0.02
Duration (per 6 months)	1.25	1.0–1.5	0.02
Extraction plan (yes)	1.34	0.8–2.0	0.18

Subgroup and sensitivity analyses yielded results consistent with the primary findings, confirming the robustness of the association between aligner therapy and reduced root resorption.

## Discussion

The present CBCT-based retrospective study compared apical root resorption in patients treated with clear aligner therapy and fixed orthodontic appliances. To the authors’ knowledge, this is among the limited CBCT-based comparative studies incorporating both volumetric and linear assessments with mixed-effects modeling for clustered tooth-level data in evaluating appliance-related root resorption.

The findings demonstrated that clear aligner therapy was associated with significantly lower root volume loss and reduced root length shortening compared with fixed appliances. This difference was most pronounced in anterior teeth, particularly incisors, which are known to be more susceptible to OIEARR. In addition, the prevalence of clinically significant resorption (≥ 2 mm) was lower in the aligner group, supporting a potential biological advantage of aligner-based mechanics.

These findings are consistent with previous CBCT and radiographic studies reporting reduced root resorption with clear aligner therapy compared to fixed appliances [9–11]. Recent investigations using three-dimensional imaging have shown that aligners are associated with less severe volumetric and linear root changes, especially in maxillary incisors, where orthodontic forces and torque demands are highest [9–11]. Although statistically significant, the absolute differences in root length reduction were modest. However, even small reductions may be clinically relevant in patients predisposed to resorption or requiring prolonged treatment. More recent CBCT-based analyses have further supported these observations, suggesting that aligner systems may exert more controlled and biologically favorable force systems than continuous arch wire mechanics [12].

The observed association between aligner therapy and lower root resorption may be partially explained by differences in biomechanics and force application. Clear aligners typically deliver intermittent, low-magnitude forces, allowing periods of periodontal ligament recovery and reduced inflammatory response. Recent systematic evidence has also explored the relationship between orthodontic appliance type and root resorption. A systematic review and meta-analysis by Singh et al. [13] reported that patients treated with clear aligners generally exhibited lower levels of external apical root resorption compared with those treated with fixed appliances. Similarly, an umbrella review by Selvaraj et al. [14] concluded that although root resorption occurs with both treatment modalities, aligner therapy may be associated with a lower risk of severe resorption in certain clinical situations. The findings of the present CBCT-based analysis are broadly consistent with these observations and further support the hypothesis that biomechanical differences between aligners and fixed appliances may influence the biological response of the periodontal ligament and root surface.

In contrast, fixed appliances often apply more continuous forces, which may lead to prolonged periodontal ligament compression, vascular compromise, and increased cementoclastic activity at the root apex. Experimental and clinical studies have shown that sustained orthodontic forces are associated with increased expression of pro-resorptive mediators such as RANKL and inflammatory cytokines, thereby increasing the risk of OIEARR [15–17].

Although the absolute intergroup differences in root length were relatively small, such reductions may be clinically meaningful in susceptible patients, particularly those with pre-existing short roots, prior history of resorption, or anticipated prolonged orthodontic treatment duration. In these cases, even small reductions in root integrity may influence treatment planning and radiographic monitoring strategies.

Treatment duration emerged as an independent predictor of root resorption in the present study, irrespective of appliance type. This finding aligns with previous reports indicating that longer orthodontic treatment increases cumulative mechanical loading and biological stress on the root surface, thereby elevating resorption risk [18–20]. Although extraction cases showed a trend toward greater resorption, this association did not reach statistical significance after adjustment, suggesting that treatment duration and force characteristics may play a more critical role than extraction status alone.

The novelty of the present study lies in its combined use of CBCT-based volumetric and linear root resorption assessment together with multilevel mixed-effects statistical modeling to account for clustering of multiple teeth within individual patients. In addition, unlike many previous studies, the present analysis simultaneously adjusted for clinically relevant confounders including tooth type, arch, extraction status, and treatment duration, thereby providing a more robust comparative evaluation of root resorption between clear aligner and fixed appliance therapy.

Several limitations should be acknowledged. First, the retrospective study design inherently limits causal inference. Second, the specific type and magnitude of orthodontic tooth movements, such as intrusion, torque, or bodily movement, could not be quantitatively assessed due to limitations in the available clinical records. These biomechanical factors are known to influence the development of apical root resorption and may partially contribute to the observed differences between treatment modalities. Third, all treatments were performed by a single experienced orthodontist, which ensured consistency in treatment protocols but may limit the generalizability of the results to broader clinical settings where operator experience varies. Finally, the type and severity of malocclusion were not systematically classified in the available records and therefore could not be included in the statistical models, which may introduce potential selection bias between treatment groups. Furthermore, CBCT imaging was limited to pre- and post-treatment assessments, preventing evaluation of the temporal progression of root resorption during active orthodontic treatment. However, repeated CBCT scans during treatment are ethically constrained due to radiation exposure, making the two-time-point design clinically appropriate.

Overall, the findings of this study support growing evidence that clear aligner therapy is associated with a lower extent and frequency of apical root resorption compared with fixed orthodontic appliances, particularly in anterior teeth. From a clinical perspective, aligner therapy may be considered a biologically favorable option for patients at increased risk of root resorption or in cases where controlled, gentle tooth movement is desired. Future prospective and randomized studies with standardized treatment protocols and long-term follow-up are warranted to confirm these results and further elucidate the biological mechanisms underlying appliance-related differences in root resorption.

## Conclusion

Within the limitations of this CBCT-based retrospective study, clear aligner therapy was associated with a lower extent and frequency of apical root resorption compared with conventional fixed orthodontic appliances. The difference was particularly evident in anterior teeth, especially incisors, while longer treatment duration was independently associated with increased resorption severity. Although the observed differences were modest, they may be clinically relevant in patients predisposed to root resorption or requiring prolonged orthodontic treatment. These findings suggest that clear aligner therapy may represent a potentially favorable treatment modality in carefully selected patients. Prospective studies with standardized protocols and detailed characterization of tooth movement are needed to further validate these associations.

## Data Availability

The data set used in the current study will be made available on request from the corresponding author.
